# Chronic crude garlic-feeding modified adult male rat testicular markers: mechanisms of action

**DOI:** 10.1186/1477-7827-7-65

**Published:** 2009-06-24

**Authors:** Imen Hammami, Souheila Amara, Mohamed Benahmed, Michèle V El May, Claire Mauduit

**Affiliations:** 1 Unité de recherche n° 01/UR/08-07, Faculté de Médecine, 15 rue Djebel Lakhdar, 1007 Tunis; Tunisie; 2 Inserm, U407, Oullins, F-69921, France; Université de Lyon, Oullins, F-69921, France; Université Lyon 1, Oullins, F-69921, France; 3 Hospices Civils de Lyon, Centre Hospitalier Lyon-Sud, Laboratoire d'Anatomie et de Cytologie Pathologique, Pierre-Bénite Cedex F-69495, France

## Abstract

**Background:**

Garlic or Allium sativum (As) shows therapeutic effects such as reduction of blood pressure or hypercholesterolemia but side-effects on reproductive functions remain poorly investigated. Because of garlic's chemical complexity, the processing methods and yield in preparations differ in efficacy and safety. In this context, we clarify the mechanisms of action of crushed crude garlic on testicular markers.

**Methods:**

During one month of treatment, 24 male rats were fed 5%, 10% and 15% crude garlic.

**Results:**

We showed that crude garlic-feeding induced apoptosis in testicular germ cells (spermatocytes and spermatids). This cell death process was characterized by increased levels of active CASP3 but not CASP6. Expression of the caspase inhibitors BIRC3 and BIRC2 was increased at all doses of As while expression of XIAP and BIRC5 was unchanged. Moreover, expression of the IAP inhibitor DIABLO was increased at doses 10% and 15% of As. The germ cell death process induced by As might be related to a decrease in testosterone production because of the reduced expression of steroidogenic enzymes (Star, Cyp11a, Hsd3b5 and Hsd17b). Evaluation of Sertoli markers showed that TUBB3 and GSTA2 expression was unchanged. In contrast, AMH, RHOX5 and CDKN1B expression was decreased while GATA4 expression was increased.

**Conclusion:**

In summary, we showed that feeding with crude garlic inhibited Leydig steroidogenic enzyme expression and Sertoli cell markers. These alterations might induce apoptosis in testicular germ cells.

## Background

*Allium sativum *(As) or garlic is a popular spice usually eaten both raw and cooked in various doses and its potential medical properties have been recognized for thousands of years [1,2]. Garlic is one of the ten commonest herbal medicines used in the United States (ranked in fifth place), according to recent sales data (for a review see, [3]). Indeed, As displays therapeutic effects such as in the treatment of hypercholesterolemia [4,5], prevention of arteriosclerosis [6,7] and some cancers [8,9], and presents anticoagulant [10,11] and antihypertensive [12] properties. For example, non-pharmacological treatment with garlic preparation is suggested to reduce blood pressure in hypertensive individuals (for a review, see [13]). The side-effects, particularly on male reproduction, of such a chronic treatment are poorly investigated. To date, it has been reported that heated garlic juice was effective in recovery of testicular function after experimental testicular hypogonadism [14] but other laboratories have reported that powder [15] or crude [16] garlic preparations impaired testicular and male reproductive tract functions. Moreover, garlic metabolites such as diallyl trisulfide have been reported to have spermicidal effects [17,18]. The mechanisms of garlic action on male reproduction function, and particularly on spermatogenesis, however, remain unknown.

In the present study, we investigated the mechanisms of action of chronic consumption of crude garlic on testicular functions. We tried to identify the cellular and molecular targets of crude garlic administrated in various doses to adult male rats.

The last step of apoptosis, before the irreversible cleavage of crucial proteins and endonuclease activation, is under the control of effector caspases named Caspase 3, 6 or 7. Caspase 3 (CASP3) has been studied extensively and is known to be synthesized as a pro-enzyme which needs cleavage to be active [for a review, see [19]). Activation and/or activity of CASP3, 7 or 9 is regulated by the Inhibitors of Apoptosis Proteins (IAPs). IAPs share a common domain known as BIR (baculovirus IAP repeat), that binds to and inhibits CASP3, 7 or 9 [20,21]. Among the IAP family are XIAP, BIRC3 (previously known as c-IAP1), BIRC2 (previously known as c-IAP2) and BIRC5 (previously known as Survivin) [22]. A third level of regulation has also been observed. IAP action can be inhibited by mitochondrial proteins such as DIABLO [23-25]. Therefore, the fate of the cell at the executioner step of apoptosis depends on the relative quantity of each actor: effector caspase; IAPs and IAP inhibitors, such as DIABLO. In addition to the caspase pathway, the AIFM1 (Apoptosis-inducing factor mitochondrion-associated 1) exists, which is a phylogenetically ancient mitochondrial inter-membrane flavoprotein endowed with the unique capacity to induce caspase-independent peripheral chromatin condensation and large-scale DNA fragmentation when added to purified nuclei [26].

## Methods

### Materials

TRIzol^®^, Moloney murine leukemia virus (M-MLV) reverse transcriptase and deoxynucleotide triphosphates (dNTPs) were obtained from Life Technologies, Inc. (Eragny, France). Protease-inhibitor cocktail, calf thymus deoxynucleotidyl terminal transferase and biotin 16-deoxyuridine 5-triphosphate (16-dUTP) were obtained from Roche Molecular Biochemicals (Mannheim, Germany). Sigma (Meylan, France) was the source for random hexanucleotides, acrylamide/bisacrylamide 37.5/1, Biomax MR films, deoxyribonuclease I (DNase I), ACTIN antibody (A5060), diaminobenzidine (DAB), nickel chloride, cobalt chloride, sodium cacodylate and Fast Red. *Taq *polymerase was purchased from Promega Corp. (Madison, WI, USA). Bovine serum albumin (BSA) was purchased from Euromedex (Souffelweyersheim, France). The SuperSignal^® ^West Pico Chemiluminescent kit was obtained from Pierce (Perbio Science, Brebières, France). CASP3 (9661), CASP6 (9762) antibodies were obtained from Ozyme (Saint Quentin en Yvelines, France). BIRC3 (sc-7943), BIRC2 (sc-7944), BIRC5 (sc-10811), active AIFM1, DIABLO (sc-12683), GATA-4 (GATA binding protein 4), AMH (Anti-Müllerian Hormone) and RHOX5 (reproductive homeobox 5, previously known as PEM) were obtained from Santa Cruz Biotechnology (Santa Cruz, CA, USA). Antibodies raised against XIAP (X-linked inhibitor of apoptosis) and against CDKN1B (cyclin-dependent kinase inhibitor 1B, previously known as p27^kip1^) were obtained from BD Sciences Pharmingen (Pont de Claix, France). GSTA2 antibody (Glutathione S-transferase alpha2, NCL-GSTa) was obtained from Novocastra Laboratories (Rungis, France). Antibody raised against TUBB3 (Tubulin beta3, clone TUJ1) was obtained from R&D Systems Europe (Lille, France). Oligonucleotide primers were purchased from Proligo SAS (Paris, France). Envision^+ ^kit (mouse or rabbit), hematoxylin and Faramount^® ^were obtained from Dako (Trappes, France). UltraProbe Basic Reagent 2 was purchased from Biomeda (Burlingame, CA). Superfrost Plus glass slides were obtained from Menzel-Glaser (Frelburg, Germany). The English edition of the manuscript was checked by a professional proofreading service (Proof-Reading-Service.org, Hertfordshire, United Kingdom).

### Plant preparation

The type of As used in the present study was "spring garlic". It has pink bulbs and is planted between December and March (according to the weather) in Tunisia and collected in July. This type of garlic contains 2.1% of proteins, 30% of carbohydrates, 1.5% of fibres, 0.2% of fat, 0.015% of vitamins and 0.7% minerals. The As plant used in this study was grown in Tunisia and purchased from a local market. Every day the garlic pellets were made by mixing peeled cloves of garlic with powdered standard rat pellet diet (Industrial Society of Food, Sfax, Tunisia) at three doses: 5%, 10% and 15%. For example, 15% pellets for one rat were prepared by mixing 4.5 g of crude garlic with 25.5 g of powdered standard diet in 5 mL of water. Cloves were crushed in distilled water in order to minimize volatile compound loss. A similar volume of water was added to the other doses [16].

### Animal treatments

A total of 24 adult male rats of Wistar strain were used for the study as previously described [16]. Control animals received standard pellet diet (0%). The other groups received diet supplemented with 5%, 10% and 15% of As. Every day, 30 g of food (garlic mixed with standard diet) was given to each rat. After 30 days of treatment the rats were sacrificed by decapitation. Testes were weighed and dissected. For each animal, the first testis was fixed in formaldehyde followed by routine paraffin embedding for TUNEL and immunohistochemical analysis; the second testis was frozen at -80°C for molecular and Western blot analyses. All studies on animals were conducted in accordance with current regulation and standards approved by the Faculty of Medicine of Tunis Animal Care Committee.

### Study design

In the present study, we investigated the effects of chronic consumption of crude garlic on testicular markers. First, apoptosis through TUNEL and CASP3 immunostaining approach was analyzed. In the following experiments, we have examined how treatment with As could induce changes in the expression of effector CASP3 and 6, of their cellular inhibitors (XIAP, BIRC2, BIRC3 and BIRC5) and mitochondrial pro-apoptotic factors (DIABLO and AIFM1). Since germ cell apoptosis might be linked to impairment of gonadotropin delivery and/or Leydig or Sertoli cell dysfunction, we have further evaluated these aspects.

### TUNEL (TdT-mediated dUTP-X Nick End Labeling)

Paraffin sections (5 μm) of formaldehyde-fixed testicular tissues were mounted onto Superfrost Plus slides. The sections were handled as previously described [27]. For each rat testis at least 100 random seminiferous tubules were numbered. The results were expressed as the apoptotic rate, i.e. the number of TUNEL positive cells per number of Sertoli cells.

### Immunohistochemistry

Paraffin sections were incubated for 20 min at 93°–98°C in citric buffer (0.01 M, pH 6) and left to cool for 20 min at room temperature. The sections were rinsed twice for 5 min in osmosed water, and washed twice for 5 min in Tris-buffered saline (TBS) containing 0.1% Tween-20. The Envision^+ ^kit was used for detection of anti-cleaved CASP3 antibody (dilution 1/50) according to the manufacturer's recommendations. The antigen-antibody complexes were stained with DAB which generated a brown color at the site of peroxidase activity. The sections were rinsed twice for 5 min in osmosed water, counterstained with hematoxylin for 5 min and mounted in Faramount^®^.

### RNA extraction and Reverse Transcription-Polymerase Chain Reaction (RT-PCR) coamplification

Total RNA were extracted from testicular tissues with TRIzol^® ^reagent according to the manufacturer's recommendations. The amount of RNA was estimated by spectrophotometry at 260 nm. The cDNAs were obtained by reverse transcription of 5 μg of total RNAs using random hexanucleotides as primers (5 μM) in the presence of dNTP (200 μM), dithiothreitol (10 mM) and M-MLV reverse transcriptase (200 U/μl) for 1 h at 37°C. For the PCR analysis, the target genes *Star *(Steroidogenesis activator protein), *Cyp11a1 *(previously known as P450 cholesterol side-chain cleavage), *Hsd3b5 *(previously known as 3-beta-hydroxysteroid dehydrogenase), *Hsd17b3 *(17-beta-hydroxysteroid dehydrogenase), *Cyp19a1 *(previously known as P450 aromatase) and *Srd5a2 *(previously known as 5alpha-reductase) were coamplified with the standard housekeeping gene *Actb *(beta-actin), in the presence of 2 μl of cDNA mixture, 0.02 U/μl of Taq polymerase, 1 μM of target primers, 0.5 to 1 μM *Actb *primers, 1.5 mM MgCl_2_, 100 μM dNTPs and 0.075 μl of [α-^33^P] dATP. The PCR amplification was performed by first heating the mixture at 95°C for 5 min followed by several cycles (see Table 1) consisting of three steps: one at 95°C for 30 sec, a step at melting temperature (Tm, see Table 1) for 30 sec and a step at 72°C for 30 sec. The PCR reaction ended with a step at 72°C for 5 min. After amplification, the coamplified PCR products for the target and the standard genes were separated onto an 8% polyacrylamide gel. The gel was dried and exposed on a phosphor screen (Packard, Meriden, CT) for about 30 min to 1 h. The phosphor screen was scanned by a cyclone phosphorimager (Packard, Meriden, CT) and the band intensities for each PCR product were quantified with OptiQuant software (Packard, Meriden, CT). The data were expressed as a target gene/*Actb *mRNA ratio. The sequences for the primers are reported in Table 1. The PCR reactions were conducted within the logarithmic phase of amplification. The PCR-amplified products were checked by direct sequencing. The RT-PCR primers were designed inside separate exons to avoid any bias caused by residual genomic contamination. Moreover, for all primers, no amplification was observed when PCR was performed on RNA preparations.

**Table 1:** Primer sequences and PCR conditions

Primer Names	Primer Sequences (Forward And Reverse)	Mealting Temperature (°C)	Cycles	PCR Product Length (Bp)
Star	5'-ATGTTCCTCGCTACGTTCAAG-3' 5'-CTTCCAGCCAGTGGATGAAGC-3'	57	33	695
Cyp11a1	5'-AGGTGTAGCTCAGGACTTCA-3' 5'-AGGAGGCTATAAAGGACACC-3'	64	24	399
Hsd3b5	5'-TCACATGTCCTACCCAGG-3' 5'-ATTTTTCAGGATGCTCCC-3'	62	26	264
Hsd17b3	5'-TTCTGCAAGGCTTTACCAGG-3' 5'-ACAAACTCATCGGCGGTCTT-3'	55	30	653
Srd5a2	5'-CAATCCTGCAAGATTCCACC-3' 5'-ATTGGTCCTTGGGTGCATTC-3'	52	26	380
Cyp19a1	5'-GCTTCTCATCGCAGAGTATCCGG-3' 5'-CAAGGGTAAATTCATTGGGCTTGG-3'	52	32	289
Actb	5'-TTGCTGATCCACATCTGCTG-3' 5'-GACAGGATGCAGAAGGAGAT-3'	according to target gene		146

### Western blotting analyses

Proteins were obtained from testicular tissues as previously described [16]. Proteins (15 – 40 μg) were resolved on a 10%–15% sodium dodecyl sulfate/polyacrylamide gel. Proteins were electrophoretically transferred to a nitrocellulose membrane using 25 mM Tris -185 mM glycine buffer (pH 8.3) containing 20% methanol at a constant voltage of 100 V for 1 h. After transfer, the membranes were incubated in a blocking buffer (TBS containing 1% of BSA and 0.1% Tween-20) for 2 h at room temperature. The membranes were rinsed three times with TBS/0.1%Tween-20 for 10 min each, and incubated with the first antibody (in TBS containing 1% of BSA) overnight at 4°C. The antibodies were diluted as follows: 1/100 for GATA-4 and AMH; 1/200 for RHOX5 and CASP3; 1/400 for BIRC3 and BIRC5; 1/500 for CDKN1B; 1/600 for DIABLO; 1/1,000 for CASP6 and GSTA2; 1/2,500 for BIRC2; 1/3,000 for TUBB3 and 1/6,000 for XIAP. The protein loading was checked by probing the blot with a rabbit IgG anti-ACTIN antibody (1/20,000). The antigen-antibody complexes were detected with a chemiluminescent kit. The membranes were exposed on Biomax MR films. The intensity of the bands was determined with OptiQuant software. The data were expressed as a target/actin protein ratio.

### Data analysis

The results are expressed as the mean ± SD. For each condition, at least six different rats were used. A one-way analysis of variance (ANOVA) for independent groups was performed to determine whether there were differences between all groups and this was followed by the Bonferroni post hoc test at p < 0.05 to determine the significance (p < 0.05) of the differences between the pair of groups. The statistical tests were performed on StatView software version 5.0 (SAS Institute Inc., Cary, NC).

## Results

### Effects of As treatment on germ cell apoptosis

**Figure 1:**
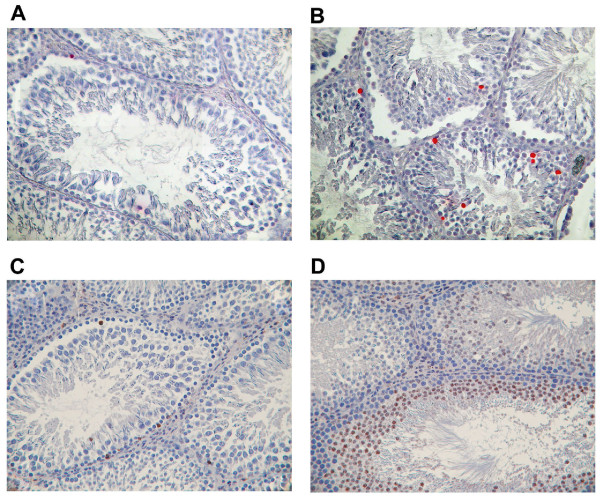
**As-induced apoptosis in germ cells (TUNEL AND CASP3 immunostaining)**. Testes from untreated rats (A, C) or adult rats fed with 15% As (B, D). Detection of apoptotic cells was conducted through the TUNEL approach (A, B) and cleaved CASP3 immunostaining (C, D) (magnification = ×200). Red staining reveals the TUNEL positive nuclei and brown staining reveals the CASP3 cytoplasm immunostaining.

**Figure 2:**
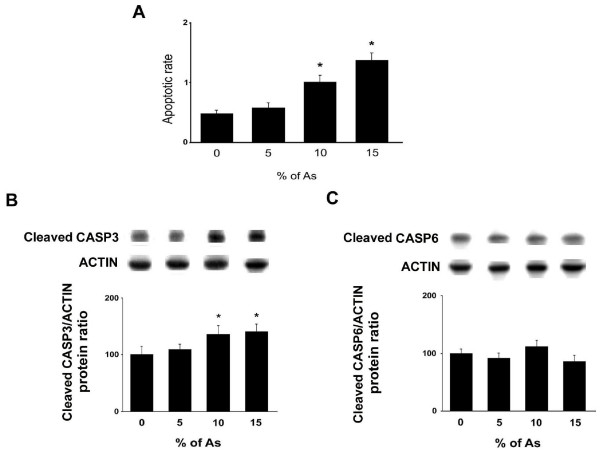
**Quantitative effects of As administration on apoptosis in the testis (TUNEL and Western blotting)**. Adult rats were untreated (0%) or fed with 5, 10 and 15% As. (A) The apoptotic rate represents the number of apoptotic germ cells (TUNEL approach) per Sertoli cells. (B) cleaved CASP3 and (C) cleaved CASP6 protein levels were analyzed through western blotting approach. The results are expressed as the mean ± SD.

Treatment with As induced a cell death process in the adult rat testis as shown by the TUNEL approach. In the control untreated animals, very few apoptotic germ cells were observed (Fig. 1A), whereas TUNEL-positive cells were identified in rat testis treated with 15% of As (Fig. 1B). These TUNEL-positive cells were mainly spermatocytes and spermatids (Fig. 1B). The number of apoptotic germ cells in rat testes increased after treatment with As in a dose-dependent manner (Fig. 2A). A significant (*p *< 0.0001) increase was observed in the rats treated with 10% and 15% of As. The cell death process induced in spermatocytes and spermatids from rats fed with As was probably an apoptotic mechanism, since an immunostaining for cleaved CASP3 was detected in these cells (Fig. 1D) while only a few stained germ cells were detected in untreated rats (Fig. 1C).

### Effects of As feeding on the executioner step of apoptosis in the adult rat testis

**Figure 3:**
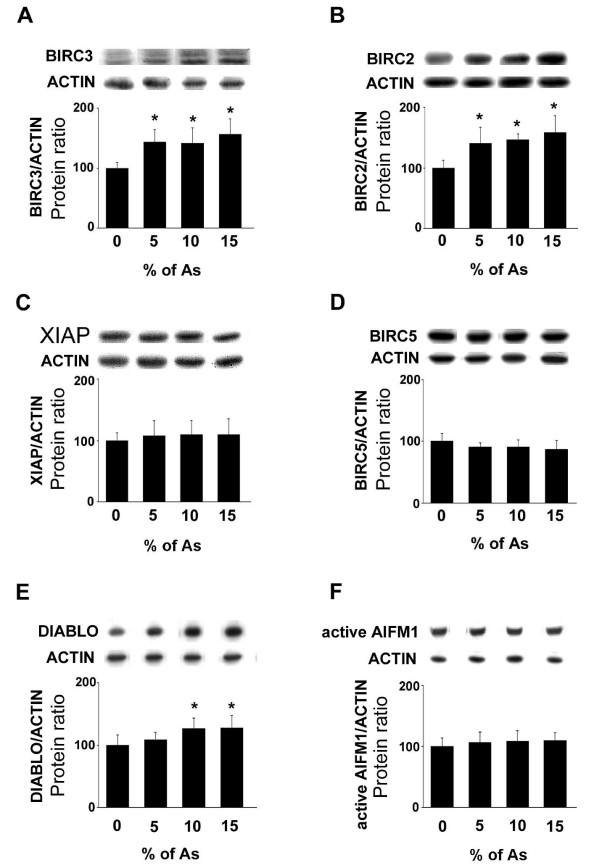
**Effects of As administration on BIRC3, BIRC2, XIAP, BIRC5, DIABLO and active AIFM1 protein levels (Western blotting)**. Adult rats were untreated (0%) or fed with 5, 10 and 15% As. (A) BIRC3, (B) BIRC2, (C) XIAP, (D) BIRC5, (E) DIABLO and (F) active AIFM1 protein levels were analyzed through western blotting approach. In the upper panels, representative autoradiograms are shown. The results are expressed as the mean ± SD.

Cleaved CASP3 expression was increased in a dose-dependent manner in the testicular tissues from rats fed with As with a significant increase at doses 10% (26%, *p *= 0.0003) and 15% (29%, *p *< 0.0001; Fig. 2B). In contrast, As-feeding did not modify the expression of cleaved CASP6 (Fig. 2C). The BIRC3 protein levels were significantly increased at doses 5% (30.5%; *p *< 0.01), 10% (29%; *p *< 0.02) and 15% (36%; *p *< 0.003) of As (Fig. 3A). Similarly, the BIRC2 protein levels were significantly increased after treatment with 5% (29%; *p *< 0.002), 10% (31.5%; *p *< 0.004) and 15% (37%; *p *< 0.0003) of As (Fig. 3B). In contrast, As-feeding did not modify the protein levels of XIAP (Fig. 3C) or BIRC5 (Fig. 3D) at the different tested doses. We then evaluated the third partner of the executioner step of apoptosis, i.e. the IAP inhibitor DIABLO. Its protein levels were increased significantly at doses 10% (21%; *p *< 0.02) and 15% (21.5%; *p *< 0.02) (Fig. 3E). The apoptotic cell death process can also be induced by a caspase-independent pathway represented by AIFM1. In the testicular tissue from rats fed with As, the expression of active AIFM1 protein (Fig. 3F) levels was unchanged at the different tested doses.

### Effects of treatment with As on plasma hormone levels

In a previous report, we showed that As significantly decreased plasma and intratesticular testosterone levels, while a significant increase in LH levels was observed, but the FSH was not investigated [16]. We show here that FSH plasma levels were significantly decreased (about 19.7%) at doses 10% (without As 12.7 ± 1.91 ng/mL; vs. with 10% As 10.22 ± 1.6 ng/mL; *p *= 0.03) and 15% (10.18 ± 1.17 ng/mL; *p *= 0.04) of As.

### Effects of treatment with As on Leydig cell steroidogenic enzyme expression

**Figure 4:**
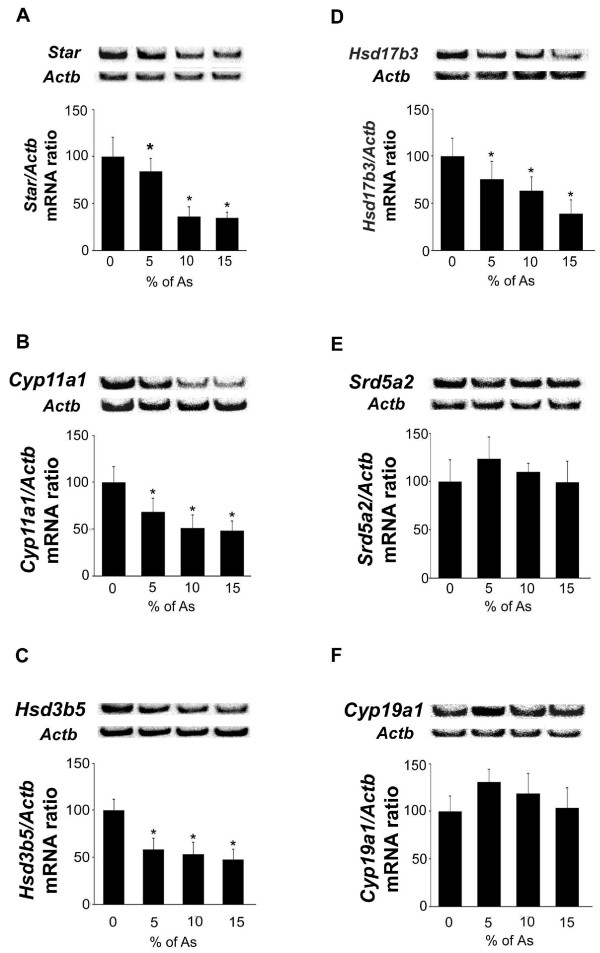
**Effects of treatment with As on Leydig cell enzymes expression (RT-PCR)**. Adult rats were untreated (0%) or fed with 5, 10, 15% As. mRNA levels for (A) *Star*, (B) *Cyp11a1*, (C) *Hsd3b5*, (D) *Hsd17b3*, (E) *Srd5a2 *and (F) *Cyp19a1 *were evaluated. In the upper panels representative autoradiograms are shown. The results are expressed as the mean ± SD.

The mRNA levels for *Star*, *Cyp11a1*, *Hsd3b5 *and *Hsb17b3 *were decreased in a dose-dependent manner (Fig. 4). The *Star *mRNA levels were significantly decreased (Fig. 4A) in testicular tissue from rats treated with 5% (16%; *p *= 0.02), 10% (64.5%; *p *< 0.0001) and 15% (66%; *p *< 0.0001) of As. Similarly, Cyp11a1 (*p *< 0.0001, Fig. 4B) and *Hsd3b5 *(*p *< 0.0001, Fig. 4C) mRNA levels were significantly decreased at the different doses of crude garlic tested. The *Hsb17b3 *mRNA levels were significantly decreased at doses 5% (25%; *p *< 0.004), 10% (37%; *p *< 0.002) and 15% (61%; *p *< 0.0001) of crude garlic (Fig. 4D). In contrast, the *Srd5a2 *(Fig. 4E) and *Cyp19a1 *(Fig. 4F) mRNA levels were unchanged after garlic treatment.

### Effects of As on Sertoli cell markers

**Figure 5:**
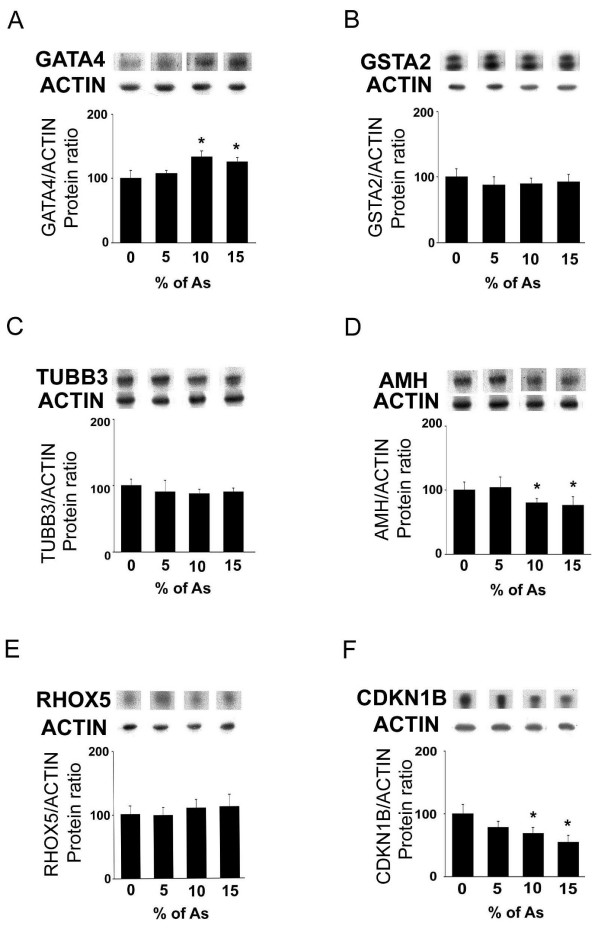
**Effects of treatment with As on Sertoli cell markers' expression (Western blotting)**. Adult rats were untreated (0%) or fed with 5, 10, 15% As. Protein levels for (A) GATA-4, (B) GSTA2, (C) TUBB3, (D) AMH, (E) RHOX5 and (F) CDKN1B were evaluated. In the upper panels representative autoradiograms are shown. The results are expressed as the mean ± SD.

Some Sertoli markers such as GATA-4, GSTA2, TUBB3, AMH, RHOX5 and CDKN1B were evaluated (Fig. 5). Expression of GATA-4 protein levels was significantly (*p *< 0.0001) increased at 10% and 15% doses of crude garlic (Fig. 5A). In contrast, GSTA2 (Fig. 5B), TUBB3 (Fig. 5C) or RHOX5 (Fig. 5E) expression was unchanged at different doses of As tested. The expression of AMH protein levels was significantly decreased after treatment with 10% (19.3%; *p *= 0.007) and 15% (24.1%; *p *= 0.001) of As (Fig. 5D). Similarly, CDKN1B expression was significantly (*p *< 0.0001) decreased at doses of 10% (31.3%) and 15% (45.6%; Fig. 5F).

## Discussion

Garlic has acquired a reputation as a formidable prophylactic and therapeutic medicinal agent over the centuries and many favorable experimental and clinical effects of the consumption of garlic, in different types of preparations (crude, powder...) have been reported (including cardiovascular diseases, stimulation of immune function, detoxification properties... for a review, see [3]). It has long been known, however, that the extraction process can increase potency compared with the crude plant. In the present study, the chemical analysis of the crude garlic used was not achieved, but Shukla and colleagues have quantified the concentration of the different compounds of garlic [9]. In this context, administration of garlic preparation to prevent hypercholesterolemia or arteriosclerosis might have side-effects on other organs. In terms of testicular functions, garlic or its metabolites have been studied as a protective adjuvant to different types of toxins [28]. Indeed, induction of testicular hypogonadism by heat is prevented in part by different types of garlic preparation (raw, heated garlic juices, dehydrated garlic powder of the more potent aged garlic extract [14]. Aqueous extract of garlic [29] or the metabolites diallyl sulfide [30] and diallyl tetrasulfide [31] offer a protection against cadmium-induced testicular damages. Garlic is also effective in restoring the testicular histology altered by EDTA [32]. The antioxidant activities of garlic extract were shown to decrease the toxic effects of free radicals induced by testicular torsion and detorsion [33]. While several studies show that As has a protective effect as an adjuvant, other studies show that it modifies spermatogenesis. Indeed, daily administration of 50 mg garlic powder over 70 days induced a spermatogenetic arrest at spermatocyte I stage [15]. Moreover, aqueous garlic extract [17] or the metabolite diallyl trisulfide [18] have spermaticidal effects. In contrast, a 90-day administration of 100 mg/kg aqueous extract of garlic has been shown to increase the number of spermatozoa [34]. In this context, to assess the positive or negative effect of garlic, it was important to analyze its action on the different testicular functions through the study of germ cell apoptosis, Leydig cell steroidogenesis and Sertoli cell markers (the present study). The present study has focused on the effects of As on testicular cells and its mechanisms of action. We showed here that oral administration of crude garlic induced germ cell death that targeted spermatocytes and spermatids, whereas spermatogonia and somatic Leydig and Sertoli cells were not affected. Given that CASP3 was immunodetected mainly in spermatocyte and spermatid cells (the TUNEL-positive germ cells) and that As treatment, at the same doses as observed for TUNEL, induced a dose-dependent increase in active CASP3 protein levels, the possibility exists that the germ cell apoptotic process could be related to CASP3 activation. The germ cell death might also, however, be related to another programmed cell death process since CASP3 activation has been associated with differentiation in some cell types [35]. In contrast, the active effector CASP6 seemed not to be involved in our model, while proCASP6 is expressed in normal germ cells [36]. While it was previously reported that chronic administration of 50 mg of garlic powder induced an arrest of spermatogenesis, our present study shows, for the first time, the involvement of an apoptotic process that targets testicular germ cells. Very few, if any, studies have reported such an apoptotic effect of As on non-tumoral cells, although it has been described in tumoral cells [37]. For example, administration of crude extract of garlic to a human colon cancer cell line induced apoptosis by increasing the levels of BAX, CYCS (previously known as cytochrome c) and CASP3 activity while it decreased the mitochondrial membrane potential [38]. More specifically, raw crushed garlic is high in allicin, a powerful bioactive compound of garlic that induced activation of CASP3, CASP8 and CASP9 and cleavage of poly (ADP-ribose) polymerase in several cancer cells [39].

Given that the balance between pro-apoptotic molecules (active effector caspase, IAPs inhibitors) and anti-apoptotic molecules (IAPs) levels determines the fate of the cells towards the executioner step of the death process [21], we have evaluated the expression of IAPs in our experimental model. BIRC2 and BIRC3 are highly expressed in rat testes [40]. BIRC3 was detected in the nucleus of B spermatogonia, spermatocytes at different stages and of somatic cells while BIRC2 was localized in the cytoplasmic compartment of spermatocytes (from stage VI pachytene onwards), spermatids (round and elongated) and Leydig cells [41]. While BIRC2 and BIRC3 play a role in the protection of germ cells from Fas-mediated apoptosis [41], their increased expression after treatment with crude garlic (the present study) suggested that germ cells are unable to inhibit CASP3 action. A potential explanation is that the high levels of DIABLO (in spermatocytes) or HTRA2 in spermatids by inhibiting the IAP action may favor the activity of CASP3 in the germ cells mainly affected by apoptosis after As treatment. The other IAPs studied here were expressed for BIRC5 in spermatocytes at first meiotic prophase and highly in Leydig cells [42] or exclusively in Leydig cells (our unpublished data) and for XIAP mainly in Sertoli cells (our unpublished data). BIRC5 and XIAP levels were unchanged after As treatment, suggesting that Leydig (BIRC5) and Sertoli (XIAP) cells are not affected in terms of apoptosis by crude garlic administration. These data are in accordance with the TUNEL approach used in the present study.

DNA degradation might also be triggered by a caspase-independent pathway through the action of AIFM1. During the apoptotic process, AIFM1 is released from mitochondria and translocated into the nucleus where its DNA binding activity mediates large-scale DNA fragmentation [26,43]. Given that As treatment, at the doses used in the present study, had no effect on the expression of active AIFM1, we suggest that the caspase-dependent pathway is mainly involved in our experimental model. The next question to address was whether germ cell death induced by As was related to modifications in hormone profile and/or Leydig and/or Sertoli cell markers.

Crude garlic induced a dose-dependent decrease in plasma and intratesticular testosterone concentrations in treated rats and an increase in LH levels [16], suggesting that As targeted Leydig cells. In this context, we evaluated the different steps of testosterone biosynthesis. Conversion of cholesterol to biologically active testosterone is a multi-step enzymatic process, including *Star*, that controls the transport of cholesterol from the outer to the inner mitochondrial membrane [44], *Cyp11a1, Hsd17b3 and Hsd3b5*. Testosterone can be metabolized by *Srd5a2 or Cyp19a1*. We showed here that As alters testosterone production, since we found that *Star*, *Cyp11a1*, *Hsd17b3 *and *Hsd3b5 *mRNA levels were decreased in a dose-dependent manner. Given that testosterone protects germ cells, especially spermatocytes and spermatids, against apoptosis [45-47], its decrease induced by As treatment might be an explanation for the death of spermatocyte and spermatid cells via an apoptotic process. Interestingly, while garlic extract is known to reduce serum cholesterol levels (in humans and animals) and inhibit cholesterol biosynthesis [48] testosterone production was not related to cholesterol metabolism [16] but to steroidogenic enzyme modification (the present study).

In terms of Sertoli cells, we showed here that both hormones which regulate cellular functions are decreased, i.e. testosterone and plasma FSH levels. In addition, the germ cell number might be decreased since the number of empty seminiferous tubules increased in a dose-dependent manner [16] and it is well recognized that germ cell loss modifies Sertoli cell functions (for a review, see [49]). In this context, we evaluated several Sertoli cell markers such as TUBB3, a housekeeping gene involved in cytosqueleton network [50] and expressed exclusively in Sertoli cells (our unpublished data), or proteins known to be regulated by testosterone (GSTA2, RHOX5, AMH, CDKN1B) and FSH (AMH) or known to be involved in paracrine interactions (GATA4). TUBB3 expression was unchanged at all doses of As used, suggesting an absence of effect of crude garlic on Sertoli cell number (the present study). These data are in accordance with the absence of apoptosis in Sertoli cells discussed above. In terms of androgen-dependent genes, we showed here that two of them (GSTA2 and RHOX5) have their expression unchanged after treatment. In contrast, AMH and CDKN1B expression was decreased while GATA-4 protein expression was significantly increased after feeding with crude garlic (the present study). Given that RHOX5 and GSTA2 expression (genes that possess ARE or ARE-like sequences) was unchanged, the possibility exists that As effects on AMH and CDKN1B are not linked to testosterone modifications but rather linked to germ cell loss. Altogether, these data indicate that As modifies some Sertoli cell markers. In the present experimental model, we observed decreased levels of testosterone associated with decreased levels of FSH. As suggested, the decreased plasma FSH levels could not be accounted for a central alteration since LH plasma levels were increased. It is noteworthy that the increase in plasma LH levels observed here was higher (about double at 10% As and 3.3-fold at 15% As) than the decrease observed in FSH plasma levels (1.2-fold at 10 and 15% As). Two hypotheses, however, might explain the discrepancies observed in the plasma gonadotropin levels. First, increased estradiol production has been shown to be associated with decreased plasma FSH without effects on LH production [51]. It is likely that in our experimental model the estradiol production was not modified since in the rats fed with crude garlic, the aromatase expression was not different compared with control animals. Second, Sertoli cells produce inhibin B which inhibits FSH secretion. Inhibin B expression is stimulated by FSH or germ cells (pachytene spermatocytes, early spermatids) and inhibited by testosterone. In the present study, the possibility exists that the dramatic decrease in testosterone production induces an increase in inhibin production that in turn decreases FSH plasma levels.

Raw garlic consumption by humans ranges from one to two cloves (about 4 g) to 28 g per day (for a review, see [3]). The concentration used in the present study exceeds this amount of consumption, but various other types of garlic preparation are consumed (such as garlic powder, oil, extract or aged garlic) and the concentration in garlic active components is highly variable, particularly in powder and oil (for a review, see [3]). Moreover, the bioactive components of garlic are not fully characterized even if it is assumed that the sulfur-containing molecules are the active ones. Another point is that garlic consumption to reduce cardiovascular risk is a long-term consumption which is another potential negative effect with regard to spermatogenesis. In this context, and more widely, extrapolation from rat to human is a difficult task. It has long been known, however, that human spermatogenesis is more sensitive to stress than that of rats [52], suggesting that concentrations lower than those used in the present study might impair male spermatogenesis and, particularly, might induce azoospermia in men with low sperm count.

## Conclusion

In summary, we showed that feeding with crude fresh crushed garlic has inhibitory effects on Leydig steroidogenic enzyme expression and Sertoli cell markers. These alterations might induce germ cell death (spermatocytes and spermatids) via an apoptotic process.

## Competing interests

The authors declare that they have no competing interests.

## Authors' contributions

IH carried out the molecular and biochemical studies. SA participated in the molecular and biochemical studies. MB participated in the design of the study and coordination. MEM performed the statistical analysis and helped to draft the manuscript. CM drafted the manuscript and participated in the design of the study. All authors read and approved the final manuscript.
